# Integrative transcriptomic, evolutionary, and causal inference framework for region-level analysis: Application to COVID-19

**DOI:** 10.1038/s41525-022-00296-y

**Published:** 2022-03-22

**Authors:** Dan Zhou, Eric R. Gamazon

**Affiliations:** 1 Division of Genetic Medicine, Department of Medicine, Vanderbilt University Medical Center, Nashville, TN USA; 2 Department of Big Data in Health Science, School of Public Health, Zhejiang University School of Medicine, Zhejiang, China; 3 Vanderbit Genetics Institute, Vanderbilt University Medical Center, Nashville, TN USA; 4 Data Science Institute, Vanderbilt University Medical Center, Nashville, TN USA; 5 Clare Hall, University of Cambridge, Cambridge, UK; 6 MRC Epidemiology Unit, University of Cambridge, Cambridge, UK

**Keywords:** Genome-wide association studies, Gene expression

## Abstract

We developed an integrative transcriptomic, evolutionary, and causal inference framework for a deep region-level analysis, which integrates several published approaches and a new summary-statistics-based methodology. To illustrate the framework, we applied it to understanding the host genetics of COVID-19 severity. We identified putative causal genes, including *SLC6A20*, *CXCR6*, *CCR9*, and *CCR5* in the locus on 3p21.31, quantifying their effect on mediating expression and on severe COVID-19. We confirmed that individuals who carry the introgressed archaic segment in the locus have a substantially higher risk of developing the severe disease phenotype, estimating its contribution to expression-mediated heritability using a new summary-statistics-based approach we developed here. Through a large-scale phenome-wide scan for the genes in the locus, several potential complications, including inflammatory, immunity, olfactory, and gustatory traits, were identified. Notably, the introgressed segment showed a much higher concentration of expression-mediated causal effect on severity (0.9–11.5 times) than the entire locus, explaining, on average, 15.7% of the causal effect. The region-level framework (implemented in publicly available software, SEGMENT-SCAN) has important implications for the elucidation of molecular mechanisms of disease and the rational design of potentially novel therapeutics.

## Introduction

A novel coronavirus, Severe Acute Respiratory Syndrome Coronavirus 2 (SARS-CoV-2), has caused a global pandemic^1^, with millions of individuals infected and over one million lives claimed worldwide. The severity of coronavirus disease 2019 (COVID-19) shows substantial interindividual variability^2^, highlighting the pressing question of the major molecular and epidemiological determinants of disease presentation. The features of the host genome that increase the risk of severe COVID-19 constitute a critical public health question^3^, with important implications for our molecular understanding of a lethal disease and for the development of effective therapeutic strategies. Several recent sufficiently-powered studies reproduced the genome-wide association study (GWAS) signal on 3p21.31^4–6^, which had been linked to the risk of respiratory failure and critical illness in COVID-19 cases^3,7^. A subsequent study found that a 49.4 Kb segment (chr3: 45,859,651–45,909,024, hg19) within the locus, which harbors the sentinel GWAS variant, is inherited from Neanderthals^8^. Despite these striking results, the causal gene or genes in the locus and the phenotypic consequences of the introgressed segment are largely unknown.

The discovery of a locus associated with severe COVID-19 underscores certain fundamental and interrelated methodological issues. Key aspects of broad methodological interest for a region- or locus- level analysis of a putatively complex disease include elucidation of (a) genome function, which may be investigated through causal inference on intermediate molecular traits; (b) evolutionary history, which may stratify the genomic data according to modeled (e.g., introgression status or archaic alleles) and unmodelled sequences; and (c) phenome-scale consequence, which may underlie the adverse outcomes of the disease or indicate comorbidities. Integrating several widely-used approaches and a newly developed summary-statistics-based method, we provide a framework that integrates these key elements into a region-level analysis, leveraging the largest collection of human transcriptomes^9–11^, to gain insights into the disease’s etiology and expressivity.

**Fig. 1:**
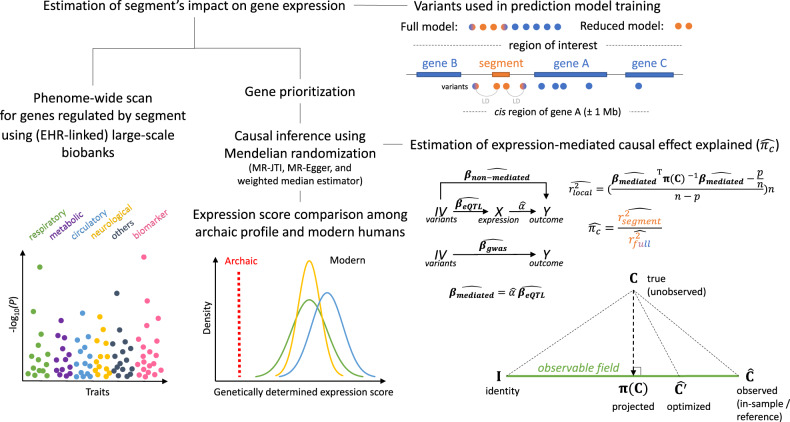
The framework. We developed an integrative transcriptomic, evolutionary, and causal inference framework for a deep region-level analysis. A segment (shown here in orange) may be a regulatory element, a stretch of DNA under positive selection, or an archaic introgressed haplotype within a potentially larger ‘region of interest’ (denoted by a broken line), which may span multiple genes and genetic variants. A segment-based analysis facilitates high-resolution quantification of the segment’s impact on (genes within) the region. The framework provides an approach to segment-specific gene expression heritability estimation using the ‘reduced model’, that is, one trained on genetic variation in the segment using the joint-tissue imputation (JTI) methodology. Region-wide gene prioritization is performed by applying the ‘full model’, that is, a model trained on all local genetic variants, to GWAS summary statistics for maximal statistical power. MR-JTI, a Mendelian randomization approach that extends JTI, estimates causal gene effects by modeling the heterogeneity due to horizontal pleiotropy and unobserved confounding. The genomic sequences are stratified according to evolutionary history (in this case, introgression status). For putative causal genes, genetically determined expression scores (GDE-scores) are compared among ‘archaic’ and ‘modern human’ genomic sequences to further quantify the evolutionary consequence of the (introgressed) segment. In addition, the proportion of expression-mediated causal effect explained by the segment $(\widehat {\pi _c})$ is quantified using a newly developed summary-statistics-based approach. Notably, we optimized the estimation of local heritability $\left( {\widehat {r_{{{{\mathrm{local}}}}}^2}} \right)$ by projecting the true (unobserved) LD matrix C to the “observable field” of covariance matrices (“Methods”) at the matrix $\pi ({{{\mathrm{C}}}})$, whose distance (mean squared error) from the true LD matrix C, denoted by $d({{{\mathrm{C}}}},\,\pi ({{{\mathrm{C}}}}))$, is minimal among the elements of the observable field. If $\pi ({{{\mathrm{C}}}})$ is the projection of the true LD matrix, ${{{\hat{\mathrm C}}}}$ is the observed (finite-sample) LD matrix (such as from the in-sample set or an external reference panel) and ${{{\hat{\mathrm C}}}}^\prime$ is some optimized version of ${{{\hat{\mathrm C}}}}$ (such as from adjustment for population heterogeneity), then $d( {{{{\mathrm{C}}}},\,\pi ( {{{\mathrm{C}}}} )} ) \le d( {{{{\mathrm{C}}}},\,{{{\hat{\mathrm C}}}}^\prime }) \le d( {{{{\mathrm{C}}}},\,{{{\hat{\mathrm C}}}}} )$. That is, we improve on the estimate of heritability by determining the unique optimal LD matrix estimator $\pi ({{{\mathrm{C}}}})$ (with minimal distance to the true LD matrix) that can be expressed as a linear combination of the identity matrix I and the observed matrix ${{{\hat{\mathrm C}}}}$ with the appropriate weights (“Methods”). To comprehensively identify the phenotypic consequences of the segment, phenome-wide scans in large-scale biobanks are conducted for genes for which the segment shows evidence of a regulatory effect. We implemented the framework in publicly available software, SEGMENT-SCAN.

This work has other broad methodological implications for studies of the genetic and molecular basis of complex traits. It presents an unbiased approach to estimating the heritability of gene expression attributable to a genomic segment (e.g., a regulatory element, a region undergoing selection, or a trait-associated locus) within a region, highlighting sources of bias for existing approaches. A segment-anchored analysis enables high-resolution quantification of its effect on genes within the region under study. This work also develops a summary-statistics-based approach to investigating, with improved causal resolution, the phenotypic consequences of a genomic region, proposing a new metric of the *proportion of expression-mediated causal effect explained*. For illustration, we apply our framework to the specific case of the COVID-19 severity associated locus (3p21.31) with the inherited archaic segment, but we emphasize the framework’s generalizability and cross-study relevance (Fig. 1).

## Results

### An overview of the framework

In this work, we developed a framework for a region-level analysis of a complex trait that performs causal inference on an intermediate molecular trait, incorporates the evolutionary history of modeled DNA sequence segment to clarify the trait’s expressivity, and evaluates a region’s broad phenotypic consequences on the human phenome (Fig. 1). We provide a software implementation, SEGMENT-SCAN, of the framework. Here, a “segment” may be a regulatory element, a stretch of DNA under positive selection, or an archaic introgressed haplotype, within a possibly larger region of interest. Leveraging the joint-tissue imputation (JTI) methodology^9^, the segment-based gene expression heritability is estimated using the “reduced model”, which includes as features the variants in the segment. A region-level gene prioritization is then performed by applying the “full model”, that is, the model trained on all local genetic variants, to GWAS summary statistics for a trait for maximal statistical power. Mendelian randomization approaches, for example, MR-JTI (an approach that estimates the gene effect size on the trait by also modeling the heterogeneity due to horizontal pleiotropy and unobserved confounding^9^), are used to increase causal support for the prioritized genes. For the putatively causal genes, since a segment (here, an introgression) may reflect the presence of an admixture (here, an ancient one) determining the local ancestry, with molecular or phenotypic consequences^12^, genetically determined expression scores (GDE-scores)^11^ are generated and compared for an ‘archaic’ genetic profile and the corresponding profile in modern human populations. In addition, the proportion of expression-mediated causal effect explained by the segment is quantified using a newly developed summary-statistics-based approach (“Methods”). To comprehensively identify the phenotypic consequences of the segment, phenome-wide scans using large-scale biobanks are conducted for the genes for which the segment shows significant evidence of a regulatory effect. Identification of potential complications or comorbidities is the goal of the phenome-wide scan. Here, we applied the framework to a COVID-19 severity related region on 3p21.31 to demonstrate the framework.

### Impact of segment on gene expression

We sought to quantify the impact of the introgressed segment(chr3: 45,859,651-45,909,024, hg19) on gene expression. For genes in the locus, we implemented JTI, a more powerful gene expression prediction approach than PrediXcan^9,10^, leveraging variants in the segment as features (“Methods”), using the 49 GTEx tissues^13^. The cross-validation performance provides an estimator of the segment-based heritability of expression that is more robust to model misspecification than the standard genome-based restricted maximum likelihood (GREML) approach (“Methods”), which assumes a polygenic architecture. In this study, for heritability, we consider only the proportion of gene expression variance explained by *cis* regulation (Fig. 1).

**Fig. 2:**
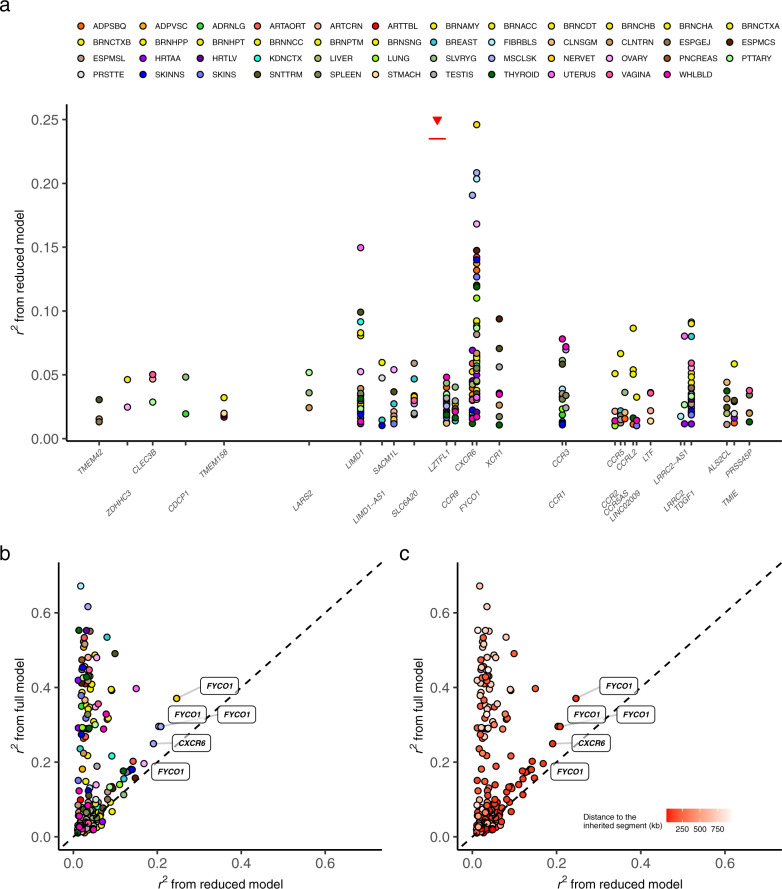
Segment-based gene expression heritability. We generated JTI gene expression prediction models in each of the 49 tissues (GTEx v8), using SNPs in the 49.4 Kb introgressed segment (whose position is marked by a red segment under a triangle in panel (**a**)) and estimated the prediction performance (*r*^*2*^) using five-fold cross-validation. Imputable genes (*r* > 0.1 and *P* < 0.05 in cross-validation; “Methods”) are shown in panel (**a**), where the *y*-axis presents the prediction performance. Panels **b** and **c** show the performan**c**e comparison between ‘full’ model and ‘reduced’ model. The ‘full’ model was trained using SNPs within 1 Mb of both sides of the gene body. The ‘reduced’ model used only SNPs in the introgressed segment (same as panel (**a**)). Panels (**b**) and (**c**) are colored according to the tissue and the distance to the segment, respectively. Highly imputable genes (*r*^*2*^ > 0.15) based on the reduced model are labeled in panels (**b**) and (**c**).

Based on the estimate from JTI, the segment explained up to 24.6%, i.e., for *FYCO1*, of the variance in gene expression in the locus (Fig. 2a). Interestingly, the protein FYCO1 was recently shown to physically interact with SARS-CoV-2’s NSP13, a helicase-triphosphatase, in a study^14^ of protein interaction map between SARS-CoV-2 and human proteins. Notably, the JTI prediction quality of the segment-based reduced model was significantly higher than the corresponding PrediXcan (which does not leverage borrowing of information across tissues) quality (*P* < 2.2e−16, Wilcoxon signed-rank test, Supplementary Table 1), with JTI improving the estimate of heritability by leveraging tissue similarity of gene expression and of its genetic regulation.

We asked whether the introgressed segment was more informative for gene expression regulation than a randomly-chosen segment of the same length (i.e., a segment with length equal to that of the introgressed one but with start position at a random position on the same chromosome). We, therefore, built a prediction model for each gene using variants in each of 100 randomly-selected segments for comparison (“Methods”). We found that the prediction performance (the square of the Pearson’s correlation *r* between the predicted expression and the observed expression) of the introgressed segment was significantly higher than the median of the prediction performance from the randomly-chosen segments (*P* = 0.038, Wilcoxon matched-pairs signed-rank test, two-sided, Supplementary Fig. 1).

### Gene expression heritability due to full model and reduced model

We sought to characterize the regulatory impact of the segment on local gene expression relative to the full *cis*-region (i.e., within 1 Mb on either side of the gene). We compared the estimate of gene expression heritability (derived from cross-validation prediction performance) from the reduced model and the full model. Two clusters of genes could be identified with the reduced model—one with a substantial reduction in performance (to near zero) and a second that lies “along the diagonal” with little performance loss (Fig. 2b). The latter set includes genes with a substantial fraction of the expression variance explained by the segment while the former includes genes that may derive much of its expression variance from outside the segment (Supplementary Table 2). Notably, the genes with heritability “concentrated” in the segment tended to be physically closer to the segment (Fig. 2c).

We investigated the impact of the segment length on the ability to maintain good prediction accuracy with the reduced model compared to the full model. This analysis also allowed us to determine the extent to which the quality of the segment calling (i.e., the accuracy of the boundary of the segment) may influence the robustness of the conclusions that can be drawn. We tested segments that include variants within 100 and 500 kb of the actual introgressed segment. As expected, the prediction performance decreased as the segment narrowed from the full *cis*-region to the actual introgressed segment. (See results in lung and whole blood in Supplementary Fig. 2a and 2b, respectively. The distributions of the prediction performance (*r*^*2*^) across all the available tissues are shown in Supplementary Fig. 2c.) The genetic variants in the segment account for only a small fraction (up to 0.035, Supplementary Fig. 2d) of the SNPs in the entire *cis*-region; nevertheless, performance degradation was observed for only a limited number of the genes in the region (consistent with Fig. 2b, c), indicating a disproportionately stronger regulatory role for the introgressed segment on local gene expression relative to the non-introgressed region.

### Region-level association test using GWAS summary statistics

**Fig. 3:**
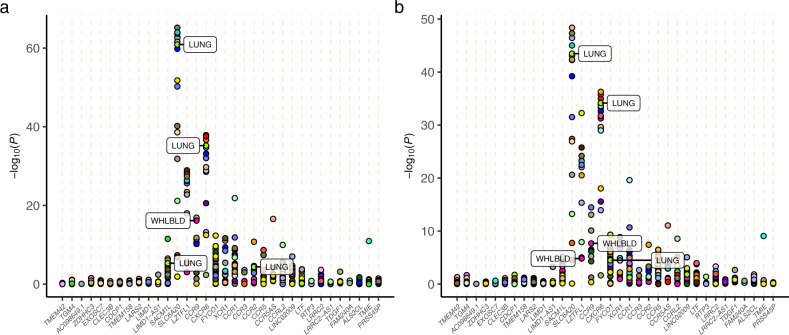
Region-level association test. Region-level association analysis of COVID-19 hospitalization (**a**) and severity (**b**) was performed on the GWAS summary statistics from COVID-19 HGI round 6. To maximize the power of the association test, we used the full model (i.e., analyzed all local variants around each gene; “Methods”) trained using JTI. The association results for the genes within 1 Mb of the introgressed segment are included in the region-level plot. Significant (*P*_FDR_ < 0.05) results observed in lung (LUNG) or in whole blood (WHLBLD) are labeled with tissue abbreviations. The positions of the genes on the *x*-axis do not represent their physical locations in the genome. (See Fig. 2a for their relative chromosomal positions.) The association results are summarized in Supplementary Table 3.

Leveraging the COVID-19 Host Genetics Initiative (COVID-19 HGI) round 6 GWAS meta-analyses^6^ for COVID-19 hospitalization (phenotype code: B2) and severity (phenotype code: A2), we performed summary-statistic-based JTI association analyses to identify hospitalization and severity associated genes in the 3p21.31 region. The sub-study information can be found in Supplementary Table 3. To maximize the power, we utilized the full model to perform the association analyses. Among the imputable genes (genes with good prediction quality; “Methods”) near the introgressed segment, we found 27 genes significantly (Benjamini–Hochberg *P*_FDR_ < 0.05) associated with the risk of hospitalization either in lung or in whole blood. *SLC6A20*, *CXCR6*, and *CCR9* were top-ranked associations (Fig. 3a). The same genes were found to be associated with COVID-19 severity (Fig. 3b). The full set of JTI association results is summarized in Supplementary Tables 4 and 5.

### Causal inference via summary statistics based Mendelian randomization

**Fig. 4:**
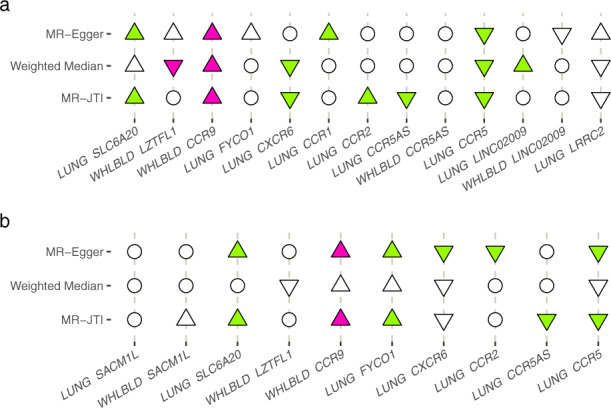
Mendelian randomization identifies candidate causal genes. MR-JTI was performed for the JTI significant (*P*_FDR_ < 0.05) genes for COVID-19 hospitalization (**a**) and severity (**b**) using the GWAS summary statistics from COVID-19 HGI round 6. Here, we provide visualization for the results derived from lung (LUNG) or whole blood (WHLBLD). Mendelian randomization results from MR-Egger, Weighed Median estimate, and MR-JTI are shown. The plot illustrates convergence of causal inference and concordance of direction of effect from the various Mendelian randomization approaches. For example, *CCR9* in whole blood showed significant causal effect and concordant direction from all three Mendelian randomization methods (panel **a**). Hollow circle, hollow triangle, and solid triangle denote non-significant, only nominally significant, and significant (i.e., after Bonferroni correction) results. Up (positive) and down (negative) triangles indicate the direction of the estimated effect sizes.

To further prioritize causal gene effects on COVID-19 severity, we applied our MR-JTI methodology^9^. MR-JTI is a two-sample Mendelian randomization approach for causal inference. Here the “exposure” is gene expression, and the “outcome” is COVID-19 severity or COVID-19 hospitalization. Summary association results for the exposure and outcome were obtained from GTEx v8 and COVID-19 HGI, respectively. Given the strong possibility of the presence of invalid instrumental variables (IVs) in the region, MR-JTI models the heterogeneity of IVs and provides a more accurate estimate of causality (see Supplementary Fig. 3 and “Methods” for comparison with the conventional inverse-variance weighted [IVW] method). In this context, the heterogeneity of IVs may be due to horizontal pleiotropic effects and unobserved confounding factors. MR-JTI was performed on genes (in lung and whole blood) with significant signals (*P*_FDR_ < 0.05) from the JTI association analysis of the COVID-19 HGI GWAS summary statistics. Six genes (namely, *SLC20A6*, *CCR9*, *CXCR6*, *CCR2*, *CCR5*, and *CCR5AS*) were significant from the MR-JTI analysis after Bonferroni correction (Fig. 4a), indicating causal support for these genes on COVID-19 hospitalization. Similarly, MR-JTI showed causal support for *FYCO1* in lung on COVID-19 severity (Fig. 4b). Mendelian randomization results from MR-Egger and weighted-median estimator were also generated using the same source data as MR-JTI (see Fig. 4, Supplementary Tables 6 and 7).

### Quantifying proportion of expression-mediated causal effect

For the MR-JTI significant genes, we further asked to what extent the gene causal effect (on the trait) was driven by the introgressed segment, quantifying the *proportion of expression-mediated causal effect* explained, π_*c*_. The statistic π_*c*_ is a ratio of estimated expression-mediated causal effects, which is calculated using a new summary-statistics-based approach (“Methods”).

We evaluated the methodological implications of our approach. Local heritability ($\widehat {r_{{{{\mathrm{local}}}}}^2}$) estimation is dependent on the LD matrix (“Methods”), which is typically estimated from the sample dataset (either in-sample or a reference panel) with finite sample size. Minimizing the distance (mean squared error) of the sample LD matrix ($\widehat C$) to the true LD matrix (C) is one way of optimizing the estimate of heritability. Use of a non-optimal LD matrix can substantially inflate the estimate of heritability. Towards this end, we obtained the unique optimal LD matrix estimator π(C) from projecting the true matrix to the “observable field” (Fig. 1 and “Methods”). Using simulations at various assumed levels of local heritability and informed by empirical genomic data (“Methods”), we confirmed that the local heritability estimated from the projected LD matrix π(C) is less biased than that estimated from the sample (e.g., external-panel-based) LD matrix Ĉ (Supplementary Fig. 4).

The inflation in the heritability estimate may also result from a genome-wide (global) approach such as LD Score regression^15^ (under a polygenic architecture). Comparison of the LD scores (calculated for a variant as the respective row sum of the LD matrix) between the original (unadjusted) LD matrix and the projected (optimal) LD matrix revealed overestimation of heritability (range: 0.4–17.1%, mean: 4.9%, Supplementary Fig. 5) with the use of the original LD matrix. Taken together, these results show that the projection matrix approach is broadly applicable, including for unbiased genome-wide heritability estimation.

We applied the optimized local heritability estimation to the seven potentially causal genes (in lung or whole blood; see Fig. 4) for either COVID-19 hospitalization or severity. On average, the segment explained 15.7% of the expression-mediated causal effect among the seven genes (Supplementary Tables 8 and 9). Notably, the concentration of expression-mediated heritability (“Methods”) was much higher (0.9–11.5 times) for the segment than the entire cis-region (Supplementary Tables 8 and 9).

### Regulatory divergence due to the segment

Paabo et al. showed that individuals with the introgressed segment are more likely to develop severe COVID-19^8^. However, the mechanism and the effector genes are unknown. To identify potentially mediating genes, we generated GDE-scores in five modern human populations (1000 Genomes project phase 3) and an approximately 122,000-year-old Altai Neanderthal sample (“Methods”), using the JTI-trained models. We emphasize that the GDE-score for a gene is not a substitute for an extinct hominin’s level of gene expression (which cannot be directly accessed), but the score allows us to stratify the genetically determined effect of a DNA sequence according to the sequence’s evolutionary history similar to local ancestry based stratification of gene expression^12^. The GDE-score for the “archaic” genetic profile provides a way to evaluate the gene expression determined by the introgressed segment in modern human populations as a function of the distance to the archaic profile. For a given gene, its JTI model was trained on genetic variants that fall naturally into categories based on their evolutionary histories, but with the effects of archaic-ancestry-specific variants remaining unmodeled^16^ (Supplementary Fig. 6). We emphasize that differences in the GDE-score reflect differences in genetic regulatory effects rather than a difference in overall expression^11^. The analysis of the difference in the GDE-scores between the archaic profile and modern human populations was performed for the Mendelian randomization-significant genes that had passed Bonferroni correction (from MR-JTI, MR-Egger, or weighted median estimator). To generate the distribution of GDE-score in modern humans for comparison with the archaic profile, we included only genes with at least two JTI model predictor SNPs available in the archaic genome.

**Fig. 5:**
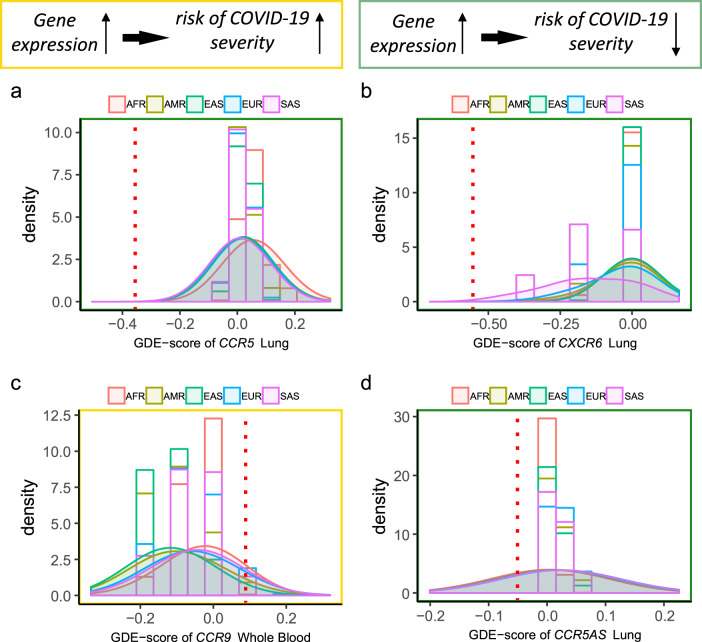
The distribution of GDE-scores in modern human populations and a carrier of an archaic genomic sequence. We applied the gene expression prediction models in the region to 2504 subjects from the 1000 Genomes project phase 3 to generate tissue-level GDE-scores for these subjects. The distributions of the GDE-scores for the five modern human populations (AFR, AMR, EAS, EUR, and SAS) are displayed for genes with significant causal effect on either COVID-19 hospitalization or severity (from the Mendelian randomization analyses), including *CCR5* (**a**), *CXCR6* (**b**), *CCR9* (**c**), and *CCR5AS* (**d**). We also generated a gene’s GDE-score in each tissue for the Altai Neanderthal genomic sequence, which is represented here by the red dash line. As illustrated in the top of the figure, the direction of effect, as estimated from MR-JTI, for the gene on COVID-19 severity is labeled by color. A yellow or green border denotes that greater genetically determined expression increases or decreases risk of severe COVID-19, respectively. Taking panel (**a**) as an example, compared with modern human genomes, the archaic sequence had a lower GDE-score for *CCR5* in lung. Given the Mendelian randomization-based finding that decreased expression of the gene increased risk of severe COVID-19, we can infer that the carriers of the archaic genomic segment would be predisposed to developing severe COVID-19.

Among the putative causal genes for either COVID-19 hospitalization or severity, the archaic sequence-based GDE-scores for *CCR5* in lung was extreme relative to modern human populations (Fig. 5a). The cross-population similarity of the GDE-score distributions for the gene in these tissues in modern humans makes the significant regulatory divergence for the archaic genomic sequence striking. Since lower expression of *CCR5* increased the risk of severe COVID-19, as estimated from the Mendelian randomization analyses, the significant difference in GDE-score indicates that carriers of the introgressed segment would have increased predisposition to severe COVID-19. A similar pattern was observed for *CXCR6* in lung, indicating that carriers of the introgressed segment have increased risk of severe COVID-19. However, in lung, *CCR9* and *CCR5AS* showed similar GDE-score profiles across modern human populations and in a carrier of the archaic genomic sequence (Fig. 5c, d).

### Phenomic scan to identify complication etiologies and comorbidities

To evaluate the broad phenotypic consequences of the introgressed segment, we performed region-level analyses of the list of genes that are well imputed by the segment (Supplementary Table 1).

**Fig. 6:**
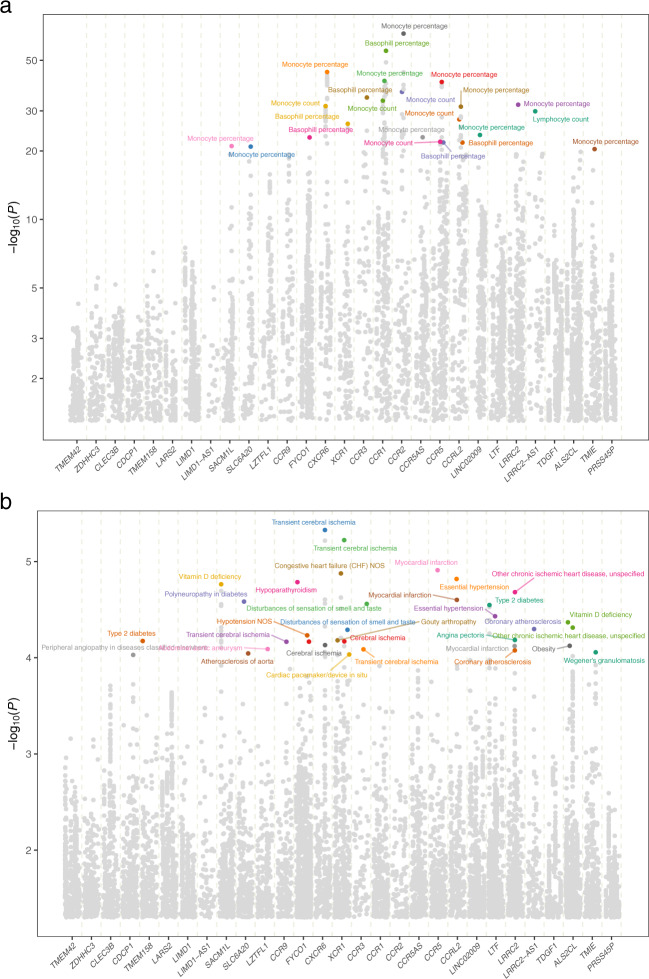
Region-level Manhattan plot of phenome-wide gene-level associations in the locus. JTI signals for phenome-wide traits, including **a** blood cell traits and **b** neurological, respiratory, circulatory, and endocrine/metabolic disorders, were observed in the region near the introgressed segment (within 1 Mb). Only genes imputable by the segment were included in this analysis. The genes are ordered by their physical positions. The minus log *P* value from the association between the genetically determined expression and the traits is shown. For each gene, only the most significant tissue is labeled. **a** For blood cell traits, highly significant results (*P* < 1.0e−20) are labeled by the trait names. **b** For potential adverse outcomes, nominally significant results (*P* < 1.0e−4) are labeled by the trait names. Additional details on the association results can be found in Supplementary Table 11 and Supplementary Table 13.

Blood cell traits are used to diagnose or monitor an infection. Considering the enrichment of immune response and chemokine-related genes in this region, we computed the gene-level JTI associations of the genes in the locus with 27 blood cell traits (Supplementary Table 10), using the GWAS summary statistics from the UK Biobank samples (see “Methods”). The severity-related genes showed significant associations with multiple blood cell traits (Fig. 6a). Notably, both *CXCR6* (*P* = 6.5e−41, lung) and *SLC6A20* (*P* = 1.4e−13, spleen; *P* = 7.1e−11, lung) were found to be significantly associated with monocyte percentage. Strong associations between the genes of the CCR family (*CCR1*, *CCR2*, *CCR3*, *CCR5*, and *CCR9*) within this locus and monocyte percentage, monocyte count, and basophil percentage were detected in multiple tissues, including whole blood and lung (Fig. 6a and Supplementary Table 11). Moreover, *CCR1*, *CCR3*, and *CCR5* were found to be associated with platelet distribution width in multiple tissues, including fibroblasts, subcutaneous adipose, tibial artery, and esophagus mucosa with *P*-values ranging from 4.8e−06 to 1.0e−02 (all passing the FDR correction, Supplementary Table 11). Taken together, the substantial associations between the genetically determined expression and inflammation, immune response, and coagulation-related blood cell biomarkers lend further support to the role of this locus in predisposition to COVID-19 severity.

We then asked to what extent genetically determined gene expression in the locus predisposes individuals to develop certain complications and adverse outcomes. Leveraging the medical phenome in the UKB, we performed a region-level phenome-scale scan across neurological, respiratory, circulatory, and endocrine/metabolic disorders (253 binary traits in total, Supplementary Table 12), limiting the analysis to the genes imputable by the segment. However, given the limited effective sample size (range: 204–251,681, Supplementary Table 12) and the large number of association tests, we emphasize that these promising results on potential complications (Fig. 6b and Supplementary Table 13) will require systematic replication in much larger datasets. Transient cerebral ischemia, myocardial infarction, and essential hypertension were found to be nominally associated with the genes in this region. Decreased genetically determined *XCR1* in esophagus mucosa was found to be nominally associated with increased risk for disturbances of sensation of smell and taste (*P* = 5.1e–05), although the significance did not survive multiple testing correction. Notably, decreased genetically determined *XCR1* in esophagus mucosa was also associated with a higher risk for severe COVID-19, indicating a potential pleiotropic effect of the gene. Taken together, these associations, which are examples among others with the same level of significance, suggest that dysregulation of genes in this locus may result in adverse outcomes and potential complications of severe COVID-19 (Supplementary Table 13).

## Discussion

Here we develop an integrative framework for the locus-specific analysis of genome function, evolutionary history, and phenome-scale impact. We build on our JTI (with its improved performance over conventional transcriptome-wide association studies) and causal inference (to account for the presence of horizontal pleiotropy or unmeasured confounding effect) methodology^9^. The framework inherently comes with a segment-based gene expression heritability estimation approach where a segment may be a regulatory element, a region under positive selection, or a trait-associated locus. Furthermore, the framework develops a new summary-statistics-based approach to estimate a metric, namely, the *proportion of expression-mediated causal effect explained*, that can be used to quantify causal mechanisms in a genomic region for a general complex disease or trait. Focusing on the introgressed segment as an application, we estimated the segment-based heritability of gene expression in the larger locus, performing a comparison of the full model and the reduced model. We prioritized genes associated with COVID-19 severity using the region-wide association test followed by several Mendelian randomization approaches (including MR-JTI). Potential complications, which implicate key biological processes underlying the infection phenotype, were identified by a phenome-wide scan for the genes regulated by the introgressed segment.

The genetic architecture of gene expression is characterized as sparse, with a small number of variants with disproportionately large effect (relative to expected from a polygenic model). We used the prediction performance ($\widehat {r_{g,s}^2}$) (for the gene *g*, of the test segment *s*, Eq. 7), which is derived from a cross-validated (additive and sparse) model of gene expression, as an estimate of the segment-specific heritability. In our application to the introgressed segment within the 3p21.31 locus associated with severe COVID-19, although the segment spans only 49.4 Kb, the genetic variants in the segment were found to explain a substantial proportion of gene expression for the neighboring genes, indicating a strong regulatory role for the segment.

An extension of PrediXcan, JTI borrows information across tissues and substantially improves gene expression prediction performance^9^. The increased power of JTI may enhance drug target discovery and improve drug repurposing efforts. By estimating the heterogeneity due to horizontal pleiotropy and unobserved confounding, MR-JTI further prioritized several genes near the introgressed segment in the associated locus as potentially causal. Importantly, we provide strong support for the regulatory role of the introgressed segment for the putatively causal genes.

We previously trained prediction models using only the (GTEx) individuals with no Neanderthal ancestry in a gene’s regulatory region and applied the models to (GTEx) individuals with Neanderthal ancestry^11^. Only a small reduction in prediction accuracy for the individuals with Neanderthal ancestry was observed relative to the models built without filtering by archaic ancestry^11^. Comparing the GDE-score of an archaic profile with the distribution in modern human populations, we found supportive evidence that the Neanderthal alleles conferred a greater predisposition to severe COVID-19. For carriers of the archaic segment, the higher risk of severe COVID-19 was driven mainly by the genetic regulation of the expression of *CCR5* and *CXCR6* in lung.

The region-level analysis prioritized *SLC6A20, CXCR6*, and the *CCR* family (*CCR5* and *CCR9*). Functional interaction between SIT1 (the protein encoded by *SLC6A20*) and ACE2 has been reported by Vuille-dit-Bille and colleagues^17^. Exploited by SARS-CoV-2 (and a SARS-CoV-2-like virus), ACE2 is a co-receptor important for viral intracellular entry into the lung and brain^18–20^. The chemokine receptor coding gene, *CXCR6*, plays a key role in NK cell-mediated memory of haptens and viruses^21^. The *CCR5* encodes the protein which belongs to the beta chemokine receptor family of integral membrane proteins^22^. A recent study showed that anti-CCR5 humanized monoclonal antibody restored CD8 counts in COVID patients, indicating CCR5 as a therapeutic target for COVID-19^23^. The chemokine receptor *CCR9* plays an important role in regulating the development and migration of T lymphocytes^24^. By utilizing CRISPR/Cas9 mediated genomic deletion, Yao et al. identified CCR9 as a potential target gene of the 3p21.31 locus for COVID-19 severity^25^.

The region-level analysis of blood cell traits further supports the connection between these genes and inflammatory traits. In addition, biomarkers for coagulation-related traits were found to be associated with the genetically determined expression of several genes in the CCR family, which show substantial genetic control by the segment. Notably, the relevance of fibroblasts^26^ and subcutaneous adipose tissue^27^, where the association signals were observed, for coagulation-related traits finds support in previous studies. Leveraging disease phenotypes in the UK Biobank, we identified potential comorbidities and complications for the region. Notably, decreased genetically determined *XCR1* in esophagus mucosa was found to be associated with increased risk for both severe COVID-19 and “disturbances of sensation of smell and taste”, which had been reported as comorbidities in 41.0 and 38.2% cases, respectively, in a previous study^28^. The protein encoded by *XCR1* is a chemokine receptor for XCL1 and XCL2 (lymphotactin-1 and -2). *XCR1* has been studied mostly in dendritic cell-based cancer immunotherapy^29^, while its role in olfactory and gustatory dysfunction is unknown. Clearly, a larger sample size and more comprehensive replication (in additional external datasets) will be required for more definitive conclusions due to the multiple comparison burden. Nevertheless, these gene-level associations can be the basis for interrogating the downstream consequences of severe COVID-19 on the broader human disease phenome and, potentially, for designing effective therapeutic strategies.

Here, we treated the gene as the basic unit for causal inference (treating its expression as the “exposure” within a Mendelian randomization framework), which is to be contrasted with fine-mapping of causal variants. To date, only limited fine-mapping of causal variants has been performed for COVID-19 severity^3,30,31^. Compared with variant-level fine-mapping, the gene-level causal inference has some desirable features, including (1) the relevance of the gene (and ease of use) as a target for drug development and repurposing; (2) increased statistical power for causal inference from leveraging multiple instrumental variables; and (3) greater portability across ethnic groups^32^. Our approach also differs from colocalization, which tests for shared causal variants for expression and the phenotype. In the Mendelian randomization framework (MR-JTI), for a gene to be causal for a phenotype, having shared causal variant effects is not enough. Clearly, the gene-level analysis does not capture coding mechanisms and other non-expression-mediated causal effects. However, we provide a framework for estimating the expression-mediated causal effect using summary statistics for downstream functional studies.

This study has several caveats and limitations. Firstly, without modeling low-frequency genetic variants, the regulatory effect of the introgressed segment may be underestimated. Low MAF variants are not very informative given the current sample sizes of available reference datasets. Secondly, although the latest GTEx dataset is a broad collection of tissues and cell types, the causal cell type(s) may be missing, or only partially represented, in the available tissues and cell types. Thus, the “tissues” in this study denote a proxy for the causal tissue(s) or cell type(s). Finally, we are unable to model archaic-ancestry-specific regulatory effects, i.e., both the non-introgressed, archaic-ancestry-derived alleles and the ancestral alleles now fixed on the modern human lineage. However, our interest here is not in predicting the transcriptome of an archaic genome (which is not available), but the effect of an introgressed segment in modern human populations.

In summary, we developed an integrative, genetics-anchored framework for a deep region-level analysis of a complex trait, which performs causal inference on an intermediate molecular trait, incorporates the evolutionary history of modeled DNA variation, and evaluates the phenome-scale impact of the implicated locus. Applying the framework to the COVID-19 severity associated locus with an archaic introgressed segment, we provided causal support for multiple genes and identified several genetically-supported adverse outcomes.

## Methods

### Estimating the segment-based heritability of gene expression

We estimated the heritability of gene expression due to a genomic segment, using a sparsity-regularization and cross-validation-based methodology. This approach, as we show below, is more robust to model misspecification than the widely used mixed model^33^ and is suitable for gene expression.

### Gene expression model building

Suppose *g*_1_, *g*_2_, …, *g*_*n*_ are *n* tissue-gene pairs of expression measurements for a given gene. We aim to find a near-optimal set of variants in the segment with effect size vector $\hat \beta$, the ‘JTI model’^9^, assuming additivity of effect:

1 $$\hat \beta = \mathop {{{{{\mathrm{argmin}}}}}}\limits_\beta (1/2)\mathop {\sum }\limits_{i = 1}^n w_i\left( {g_i - x_i^T\beta } \right)^2 + \lambda \left( {\left( {\frac{{1 - \alpha }}{2}} \right)\Vert\beta\Vert_{2}^{2} + \alpha \Vert\beta\Vert_{1}} \right)$$

The *n* × *p* matrix $[x_1,\,x_2,\, \ldots ,\,x_n]^T$ is the feature matrix (of genetic variants). The *w*_*i*_ is the weight, generated from hyper-parameter tuning, on the *i*th observation from the tissue similarity matrix. The JTI model thus leverages the similarity in transcriptional regulation profile. JTI can be extended to leverage a *d*-dimensional similarity vector (*d* ≥ 1) by incorporating several layers of epigenomic datasets, as we previously described^9^. The *L*_1_ penalty in the objective function enforces sparsity (consistent with the genetic architecture of gene expression) while the *L*_2_ penalty promotes grouping effect. Here *α* encodes the relative weight of the two penalties; we assumed *α* = 0.50. Given a test tissue, when tissue sample pairs from a different tissue are assigned weight 0 while those in the test tissue are assigned weight 1 in the loss function in Eq. 1, then the resulting special instance of the optimization problem generates the single-tissue ‘PrediXcan model’.

### Cross-validation

The vector *g* of gene expression (say of dimension *n*) in each tissue can be decomposed as:

2 $$g = \left[\begin{array}{*{20}{c}} {g_{{{{\mathrm{train}}}}}} \\ {g_{{{{\mathrm{test}}}}}} \end{array}\right] = \left[\begin{array}{*{20}{c}} {s_{{{{\mathrm{train}}}}}} \\ {s_{{{{\mathrm{test}}}}}} \end{array}\right] + \left[\begin{array}{*{20}{c}} {\varepsilon _{{{{\mathrm{train}}}}}} \\ {\varepsilon _{{{{\mathrm{test}}}}}} \end{array}\right]$$

where g_*_, s_*_, and ε_*_ are the gene expression level, the genetic component, and the residual, respectively, in the training or test set (denoted here as *). For simplicity of presentation and without loss of generality, we left out the fixed effects (covariates). Assuming $\varepsilon \sim {{{\mathcal{N}}}}(0,{{\Gamma }})$ has a Gaussian distribution, the variance-covariance matrix var(*g*) can be written as^34^:

3 $${{{\mathrm{var}}}}\left( g \right) = X{{{\mathrm{cov}}}}(\beta )X^T + {{\Gamma }}$$

where cov(*β*) is the symmetric covariance matrix of the effect size vector and *X* is the *n* × *p* genotype (feature) matrix. By independence of the training and test sets, $${{\Gamma }} = \left[ {\begin{array}{*{20}{l}} {{{\Gamma }}_{{{{\mathrm{train}}}},\,{{{\mathrm{train}}}}}} \hfill & 0 \hfill \\ 0 \hfill & {{{\Gamma }}_{{{{\mathrm{test}}}},{{{\mathrm{test}}}}}} \hfill \end{array}} \right]$$, where each submatrix ${{\Gamma }}_{ \ast , \ast }$ is symmetric.

### Sampling dependence

Here we seek a theoretical formulation of the sampling dependence of the cross-validation framework. In *K*-fold cross-validation, the dataset is partitioned into *K* non-overlapping subsets (say, of the same size *n*/*K*). Let *Test*_*k*_ and *Train*_*k*_ (that is, the dataset with the elements of *Test*_*k*_ removed) be the *k*th test set and training set, respectively. For each $i \in Test_k$, we consider the “error” or residual $\varepsilon _i$, defined as the difference between the gene expression level and the estimated genetic component trained in *Train*_*k*_ for *i*. The average residual $\varepsilon = \frac{1}{n}\mathop {\sum }\limits_{i = 1}^n \varepsilon _i$ has variance given by the following expression:

4 $$\begin{array}{lll}{{{\mathrm{var}}}}\left( \varepsilon \right)& = &\frac{1}{{n^2}}{{{\mathrm{var}}}}\left( {\mathop {\sum }\limits_{i = 1}^n \varepsilon _i} \right) = \frac{1}{{n^2}}\mathop {\sum }\limits_{i,j} {{{\mathrm{cov}}}}\left( {\varepsilon _i,\varepsilon _j} \right)\\ &=& \frac{1}{n}\sigma^{2} + \left( {\frac{1}{K} - \frac{1}{n}} \right)\delta _{{{{\mathrm{within}}}}}^2 + \left( {1 - \frac{1}{K}} \right)\delta _{{{{\mathrm{between}}}}}^2\end{array}$$

where $\sigma ^2$ is the average variance of the residuals for test samples (where the average is calculated over the training sets on which the residuals depend), $\delta _{{{{\mathrm{within}}}}}^2$ is the within-fold covariance for these test samples (which may be nonzero because of the shared training set), and $\delta _{{{{\mathrm{between}}}}}^2$ is the between-fold covariance (which may be nonzero due to the fact that each $Test_k$ is a subset of $Train_l$ when $l \,\ne\, k$). We note that

5 $$\mathop {{\lim }}\limits_{n \to \infty } {{{\mathrm{var}}}}\left( \varepsilon \right) = \frac{1}{K}\delta _{{{{\mathrm{within}}}}}^2 + \left( {1 - \frac{1}{K}} \right)\delta _{{{{\mathrm{between}}}}}^2$$

### Unbiased estimator of heritability of gene expression

The expression for the variance-covariance matrix (Eq. 3) recalls the usual decomposition of variance in the standard mixed model for heritability estimation^33^. One key difference is that the mixed model fits the genetic effects $u \in {\Bbb R}^p$ as random effects:

6 $$g = a\overrightarrow 1 + Zu + \varepsilon$$

$$u \sim {{{\mathcal{N}}}}(0,\sigma _u^2I)$$

$$\varepsilon \sim {{{\mathcal{N}}}}(0,I\sigma ^2)$$

Here $\vec 1 \in {\Bbb R}^{{{\boldsymbol{n}}}}$ is a vector of ones. The variance components $\sigma _u^2$ and $\sigma ^2$ are estimated using an algorithm (e.g., restricted maximum likelihood), and the heritability estimate $\widehat {h_{MM}^2}$ is then given by the ratio $\frac{{p\widehat {\sigma _u^2}}}{{p\widehat {\sigma _u^2} + \widehat {\sigma ^2}}}$. Now the so-called Best Linear Unbiased Predictions (BLUP) derived from the mixed model is related to ridge regression^35,36^, a common regularization approach. Maximizing the posterior *P*(*u*|*g*) under a Gaussian prior is equivalent to the minimization of the ridge objective function with ridge hyperparameter $\lambda = \sigma ^2/\sigma _u^2$. Thus, mixed model parameter estimation (and thus heritability estimation under the mixed model approach) can be viewed as a type of regularization, but in contrast to regular ridge hyperparameter estimation which requires a training and validation dataset, mixed model parameter estimation is done in a single dataset.

For gene expression, we take a different approach, which relies first on regularization (Eq. 1) and then cross-validation, both of which should reduce overfitting. Let $g_{{{{\mathrm{test}}}},0}$ be the gene expression level for one random observation from the test set. The performance of the model is given by:

7 $$\widehat {r_{g,s}^2} = \frac{{{{{\mathrm{cov}}}}(g_{{{{\mathrm{test}}}},0},\widehat {s_{{{{\mathrm{test}}}},0}})^2}}{{{{{\mathrm{var}}}}\left( {g_{{{{\mathrm{test}}}},0}} \right){{{\mathrm{var}}}}(\widehat {s_{{{{\mathrm{test}}}},0}})}}$$

Here, the estimated genetic component $\widehat {s_{{\rm{test}},0}}$ comes from applying the solution to the optimization problem given by Eq. 1 to the test subject. This coefficient of determination is an unbiased estimate of the proportion of explained variation. The regularization and cross-validation approach is the core of the JTI prediction methodology, from which, therefore, an estimator of heritability of gene expression can be defined.

We also estimated the concentration of heritability, using the statistic:

8 $$\psi = \frac{{h_{g,\,{{{\mathrm{reduced}}}}}^2p_{{{{\mathrm{full}}}}}}}{{h_{g,{{{\mathrm{full}}}}}^2p_{{{{\mathrm{reduced}}}}}}}$$

where p_*_ is the number of variants in the model (reduced or full; see section “Training the full model and reduced model of gene expression” below), which measures the per-SNP heritability from the reduced model as a fraction of the per-SNP heritability from the full model.

### Estimation of region-level (local) trait heritability using summary statistics

Using the theory of quadratic forms, we previously derived a summary-statistics-based estimator of region-level trait heritability (while accounting for linkage disequilibrium [LD]; see Equations A6 and A7 in the appendix of Gamazon et al.^37^). The estimator and its variance are given by:

9 $$\widehat {r_L^2} = \left( {\frac{{\hat \beta ^T{{{\mathbf{C}}}}^{ - 1}\hat \beta - \frac{p}{n}}}{{n - p}}} \right)n$$

10 $${{{\mathrm{var}}}}\left( {\widehat {r_L^2}} \right) = \left( {\left( {1 - \frac{{p^2}}{{n^2}}} \right)^{ - 1}} \right)\frac{{\left( {1 - \widehat {r_L^2}} \right)}}{n}\left( {4\widehat {r_L^2} + 2p\frac{{\left( {1 - \widehat {r_L^2}} \right)}}{n}} \right)$$

This estimator is defined for a locus or region *L*, is approximately unbiased when in-sample LD is close to the true LD, and can be extended, via independent LD blocks, to estimate the genome-wide SNP heritability. Here *p* is the number of SNPs, $\hat \beta$ is the *p* × 1 vector of estimated effect sizes (on the GWAS trait or on gene expression, depending on context), and **C** is the *p* × *p* SNP correlation matrix. The condition $n \ge p$ is a necessary condition for **C** being invertible or having a full rank. This approach was extended by Shi et al.^38^ in Heritability Estimator from Summary Statistics (HESS) (and then by Hou et al.^39^ to biobank-scale data) with a model of genotypes in a locus as random variables and a technique to account for rank deficiency in the LD matrix (e.g., as may arise from SNPs in perfect LD). HESS replaces, in Eq. 9, ${{{\mathbf{C}}}}^{ - 1}$ by the Moore-Penrose pseudoinverse and replaces *p* by $q = {{{\mathrm{rank}}}}({{{\mathbf{C}}}})$, that is, the maximal number of linearly independent columns or the “effective number” of SNPs. Shi et al. “regularized” the external reference LD matrix to account for noise in the matrix, using principal components. Here, we extend our earlier work and Shi et al. with a theoretical and empirical investigation into a major source of bias for the estimate of heritability.

First, for illustration, we consider two SNPs that are in LD ($r^2 = \rho$), so that the assumed LD matrix is $$\left[ {\begin{array}{*{20}{c}} 1 & \rho \\ \rho & 1 \end{array}} \right]$$. The inverse of the matrix is, therefore, $$\frac{1}{{(1 - \rho ^2)}}\left[ {\begin{array}{*{20}{c}} 1 & { - \rho } \\ { - \rho } & 1 \end{array}} \right]$$. Let $\hat \beta ^T =$[$\widehat {\beta _1}\,\widehat {\beta _2}$] be the vector of estimated variant effect sizes (from GWAS). Then the estimate of heritability (Eq. 9) can be written as:

11 $$\widehat {r_L^2} = [n(\widehat {\beta _1}^2 + \widehat {\beta _2}^2 - 2\rho \widehat {\beta _1}\widehat {\beta _2}) - 2]/(n - 2)$$

Here, we note that:

12 $$\frac{{\partial \widehat {r_L^2}}}{{\partial \rho }} = - 2\widehat {\beta _1}\widehat {\beta _2}n/(n - 2)$$

which shows the change in the estimate caused by a perturbation in LD. A special instance is that of the SNPs being independent ($\rho = 0$), so that the LD matrix is the identity matrix. In this case, as $n \to \infty$, the heritability estimate approaches $\widehat {\beta _1}^2 + \widehat {\beta _2}^2$. Another special case is that of the SNPs that are in perfect LD ($\rho = 1$) so that the LD matrix is non-invertible (that is, has determinant $\left( {1 - \rho ^2} \right) = 0$). In this case, the Moore-Penrose pseudoinverse is $$\left[ {\begin{array}{*{20}{c}} {\frac{1}{4}} & {\frac{1}{4}} \\ {\frac{1}{4}} & {\frac{1}{4}} \end{array}} \right]$$ and the estimate of heritability reduces to:

13 $$\widehat {r_L^2} = \left[ {n\left( {\frac{1}{4}\widehat {\beta _1}^2 + \frac{1}{4}\widehat {\beta _2}^2 + \frac{1}{2}\widehat {\beta _1}\widehat {\beta _2}} \right) - 1} \right]/(n - 1)$$

As $n \to \infty$, this estimate approaches $\frac{1}{4}\widehat {\beta _1}^2 + \frac{1}{4}\widehat {\beta _2}^2 + \frac{1}{2}\widehat {\beta _1}\widehat {\beta _2}$, which is the square of the weighted sum of the variant effect sizes (each of weight 1/2). Since the SNPs are in perfect LD, then the estimated effects sizes should be equal: $\widehat {\beta _1} = \widehat {\beta _2} = \hat \beta$, and any difference in the estimates may be due to genotyping error.

Now, let us consider the general case of *p* variants in the region. The use of an external LD panel (which is typically smaller in sample size than a GWAS) usually leads to a lower rank of the LD matrix and thus produces an underestimation of the variance (Eq. 10 with lower *p*). However, a larger GWAS sample size leads to improved (i.e., lower) standard error (Eq. 10 with higher *n*). The ground-truth heritability $r_L^2 = \beta ^T{{{\mathbf{C}}}}\beta$ (where ${{{\mathbf{C}}}} = [C_{ij}]$ is the LD matrix) is a quadratic form with (scalar-by-matrix) derivative with respect to **C** given by the following *p* × *p* matrix (assuming a genetic architecture where the effect size *β* is not a function of the LD matrix **C**):

14 $$\nabla _{{{\mathbf{C}}}}r_L^2\left( {{{\mathbf{C}}}} \right) = \beta \beta ^T$$

We emphasize that the genetic architecture in which *β* is independent of **C** is assumed and necessary in Eq. 14. Thus, the change in the heritability due to a perturbation in LD is a function (a monomial of degree 2 for each entry in the *p* × *p* matrix) of the effect sizes in the region. A similar conclusion holds true on the relationship between the estimator and the estimated effect sizes assuming the LD estimate ${{{\hat{\mathbf C}}}}$ from an external reference panel. The *ij*th term of the derivative matrix $\nabla _{{{{\hat{\mathbf C}}}}}r_L^2\left( {{{{\hat{\mathbf C}}}}} \right)$ with respect to ${{{\hat{\mathbf C}}}}$ equals $\widehat {\beta _i}\widehat {\beta _j}\left( {\frac{n}{{n - {{{\mathrm{rank}}}}\left( {{{{\hat{\mathbf C}}}}} \right)}}} \right)$, which quantifies the change in heritability relative to a change in (external panel based) LD between the *i*th and *j*th variants. Thus, the change in the estimate of heritability (viewed as a function of the external panel LD estimate ${{{\hat{\mathbf C}}}}$, which in turn can be viewed as a perturbation of the in-sample LD $C_{ij}$) relative to the change in the in-sample LD $C_{ij}$ is:

15 $$\begin{array}{ll}\frac{{\partial r_L^2\left( {{{{\hat{\mathbf C}}}}} \right)}}{{\partial C_{ij}}} &= {{{\mathrm{tr}}}}\left[ {\nabla _{{{{\hat{\mathbf C}}}}}r_L^2\left( {{{{\hat{\mathbf C}}}}} \right)\frac{{\partial {{{\hat{\mathbf C}}}}}}{{\partial C_{ij}}}} \right] = {{{\mathrm{tr}}}}\left[\widehat {\beta _i}\widehat {\beta _j}\frac{{\partial {{{\hat{\mathbf C}}}}}}{{\partial C_{ij}}}\left( {\frac{n}{{n - {{{\mathrm{rank}}}}( {{{{\hat{\mathbf C}}}}})}}} \right)\right]\\ &= \widehat {\beta _i}\widehat {\beta _j}\left( {\frac{n}{{n - {{{\mathrm{rank}}}}( {{{{\hat{\mathbf C}}}}})}}} \right){{{\mathrm{tr}}}}\left( {\frac{{\partial {{{\hat{\mathbf C}}}}}}{{\partial C_{ij}}}} \right)\end{array}$$

where *tr* is the trace operator. This observation argues for the importance of making available not just the GWAS summary statistics, i.e., the $\widehat {\beta _i}$, but also the in-sample LD data, i.e., $C_{ij}$. We calculated the empirical distribution of $\widehat {\beta _i}\widehat {\beta _j}$ and performed simulations on the impact of the external panel (i.e., using the statistic $\left( {\frac{n}{{n - {{{\mathrm{rank}}}}\left( {{{{\hat{\mathbf C}}}}} \right)}}} \right){{{\mathrm{tr}}}}\left( {\frac{{\partial {{{\hat{\mathbf C}}}}}}{{\partial C_{ij}}}} \right)$) on the heritability estimate. For an LD-matched reference panel, the product monomials $\widehat {\beta _i}\widehat {\beta _j}$ have a major influence on the behavior of the estimate.

Note that in Eq. 9, the inverse of the true (unobserved) LD matrix **C** or the inverse of the external panel LD estimate ${{{\hat{\mathbf C}}}}$ is required. Thus, assuming the inverses exist, we obtain an expression for the difference between ${{{\hat{\mathbf C}}}}^{ - 1}$ and ${{{\mathbf{C}}}}^{ - 1}$ in terms of the difference (noise) matrix ${{\Delta }} = {{{\hat{\mathbf C}}}} - {{{\mathbf{C}}}}$:

16 $${{{\hat{\mathbf C}}}}^{ - 1} - {{{\mathbf{C}}}}^{ - 1} = - \left( {{{{\mathbf{I}}}} + {{{\mathbf{C}}}}^{ - 1}{{\Delta }}} \right)^{ - 1}{{{\mathbf{C}}}}^{ - 1}({{\Delta }}){{{\mathbf{C}}}}^{ - 1}$$

Therefore, the term on the right determines the difference in the estimate of heritability from the use of the external LD panel and the true LD information. We note that this term is a general expression that includes the special case, such as treated in Shi et al. in which the noise Δ is addressed through use of the truncated singular value decomposition (SVD) to obtain an improved estimator $\widehat {{{{\mathbf{C}}}}_{{{{\mathbf{SVD}}}}}}$. In particular, the difference $\Delta _{{{{\mathbf{SVD}}}}} = \widehat {{{{\mathbf{C}}}}_{{{{\mathbf{SVD}}}}}} - {{{\mathbf{C}}}}$ may still bias the estimate of heritability, with the residual bias given by $\left( {\frac{n}{{n - {{{\mathrm{rank}}}}\left( {\widehat {{{{\mathbf{C}}}}_{{{{\mathbf{SVD}}}}}}} \right)}}} \right)\hat \beta ^T( - \left( {{{{\mathbf{I}}}} + {{{\mathbf{C}}}}^{ - 1}{{\Delta }}_{{{{\mathbf{SVD}}}}}} \right)^{ - 1}{{{\mathbf{C}}}}^{ - 1}({{\Delta }}_{{{{\mathbf{SVD}}}}}){{{\mathbf{C}}}}^{ - 1})\hat \beta$

Here we describe how to obtain the projected matrix $\pi ({{{\mathbf{C}}}})$, which has the property that the difference matrix ${{\Delta }}_{{{{\mathbf{Projected}}}}} = \pi \left( {{{\mathbf{C}}}} \right) - {{{\mathbf{C}}}}$ is “minimal” in the sense of minimizing the expected quadratic loss:

17 $$\pi \left( {{{\mathbf{C}}}} \right) = \mathop {{{{{\mathrm{argmin}}}}}}\limits_{{{{\mathbf{C}}}}_ \ast } \,{{{\mathrm{E}}}}[\left\| {{{{\mathbf{C}}}}_ \ast - {{{\mathbf{C}}}}} \right\|^2]$$

where ${{{\mathbf{C}}}}_ \ast$ is a linear combination of the identity matrix ${{{\mathbf{I}}}}$ and ${{{\hat{\mathbf C}}}}$, the observed (in-sample or reference) LD matrix (Fig. 1). Define $\pi \left( {{{\mathbf{C}}}} \right)$ as the Ledoit-Wolf estimator, expressed as a linear combination of ${{{\hat{\mathbf C}}}}$ and ${{{\mathbf{I}}}}$ as follows:

18 $$\pi \left( {{{\mathbf{C}}}} \right) = \frac{{b^2m}}{{d^2}}{{{\mathbf{I}}}} + \frac{{a^2}}{{d^2}}{{{\hat{\mathbf C}}}}$$

where

19 $$m = < {{{\hat{\mathbf C}}}},\,{{{\mathbf{I}}}} >$$

20 $$d = \left\| {{{{\hat{\mathbf C}}}} - m{{{\mathbf{I}}}}} \right\|^2$$

21 $$b^2 = {{{\mathrm{min}}}}\left( {d^2,\,\frac{1}{{n^2}}\mathop {\sum }\limits_{k = 1}^n \left\| {X_kX_k^T - {{{\hat{\mathbf C}}}}} \right\|^2} \right)$$

22 $$a^2 = d^2 - b^2$$

Here, $<*,*>$ and $|| \!\ast \!||$ refer to the Frobenius inner product and norm, respectively, and $X_k$ is the $p \times 1$ genotype vector for the *k*th subject. Equations 17 and 18 have a Bayesian-geometric interpretation. $\pi \left( {{{\mathbf{C}}}} \right)$ reflects the combination of prior information and sample information. The prior information states that the unobserved (true) covariance **C** is on the sphere with center at $m{{{\mathbf{I}}}}$ and radius *a*. The sample information states that **C** is on a second sphere with center at ${{{\hat{\mathbf C}}}}$ and radius *b*. The combination of the two indicates that **C** is in the intersection of the two spheres, i.e., a circle with center at $\pi \left( {{{\mathbf{C}}}} \right)$.

### Comparison of local heritability estimated from the observed LD matrix and from the projected LD matrix

We performed simulations (*n* = 500) to investigate the impact of using an external reference panel on the estimate of local heritability. We leveraged the 1000 Genomes EUR dataset for realistic simulations. For each simulation, we generated 50,000 individual-level genotype^40^ data of 50 kb segments, with LD structure informed by empirically-derived segments, which were randomly drawn from chromosome 22. We assumed various levels of local heritability ($h_{{{{\mathrm{local}}}}}^2 =$ 0.01, 0.02, and 0.03). For each value of heritability, we generated the phenotype: $Y = \beta G + \varepsilon$. Here, *G* denotes the genotype in dosage (scaled to standard normal distribution) of a randomly sampled causal variant; $\beta = \sqrt {\frac{{h_{{{{\mathrm{local}}}}}^2 \times {{{\mathrm{var}}}}(Y)}}{{{{{\mathrm{var}}}}(G)}}}$ is the effect size of the causal variant; $\varepsilon$ denotes the residual term randomly drawn from a normal distribution $\varepsilon \sim {{{\mathcal{N}}}}(0,\sigma ^2)$ where $\sigma ^2 = {{{\mathrm{var}}}}\left( Y \right) - {{{\mathrm{var}}}}(\beta G)$ and $Y\sim {{{\mathcal{N}}}}(0,1)$. The marginal effect size for each of the variants on the segment was estimated. We randomly sampled 500 subjects to be used as an “external reference panel” and, in addition, calculated the observed LD matrix ${{{\hat{\mathbf C}}}}$ and projected LD matrix $\pi ({{{\mathbf{C}}}})$. The local heritability was then estimated (Eq. 9) using each LD matrix for comparison.

### Summary-statistics-based estimation of the proportion of expression-mediated causal effect explained

To estimate the extent to which the gene causal effect is driven by the segment of interest, we developed a summary-statistics-based approach using the projected LD matrix. We define a new metric $\pi _c$ to estimate the proportion of expression-mediated causal effect explained by a genomic segment using summary statistics. (To illustrate the approach, we evaluated the causal role of the introgressed segment in severe COVID-19. However, the approach can be applied more generally to GWAS summary statistics data.) Let $\hat \alpha$ be the MR-JTI estimate of the gene causal effect on the trait, which is obtained by solving an optimization problem (of predicting a variant’s GWAS effect size by its regulatory effect on the gene and its contribution to heterogeneity) (see below; Eq. 29). We consider the GWAS marginal effect size vectors, $\widehat {\theta _{{{{\mathrm{full}}}}}}$ and $\widehat {\theta _{{{{\mathrm{reduced}}}}}}$, and corresponding eQTL effect size vectors, $\widehat {\beta _{{{{\mathrm{full}}}}}}$ and $\widehat {\beta _{{{{\mathrm{reduced}}}}}}$, for the full model and reduced model, respectively, and the projected matrices ${{{\mathbf{C}}}}_{{{{\mathrm{full}}}}}^ \ast$ and ${{{\mathbf{C}}}}_{{{{\mathrm{reduced}}}}}^ \ast$ of the SNP correlation matrices ${{{\mathbf{C}}}}_{{{{\mathrm{full}}}}}$ and ${{{\mathbf{C}}}}_{{{{\mathrm{reduced}}}}}$ for the full model and reduced model, respectively. We have the following decomposition of the GWAS marginal effect size into an expression-mediated causal effect and an “indirect” component (Fig. 1):

23 $$\widehat {\theta _ \ast } = \widehat {\theta _{{{{\mathrm{mediated}}}}}} + \widehat {\theta _{{{{\mathrm{unmediated}}}}}} = \hat \alpha \widehat {\beta _ \ast } + \widehat {h_ \ast }$$

where * denotes the full or reduced model. Then we define $\pi _c$ as follows:

24 $$\pi _c = \left( {\frac{{\hat \alpha ^2\widehat {\beta _{{{{\mathrm{reduced}}}}}}^T{{{\mathbf{C}}}}_{{{{\mathrm{reduced}}}}}^ \ast \widehat {\beta _{{{{\mathrm{reduced}}}}}} - \frac{{{{{\mathrm{rank}}}}\left( {{{{\mathbf{C}}}}_{{{{\mathrm{reduced}}}}}} \right)}}{n}}}{{\hat \alpha ^2\widehat {\beta _{{{{\mathrm{full}}}}}}^T{{{\mathbf{C}}}}_{{{{\mathrm{full}}}}}^ \ast \widehat {\beta _{{{{\mathrm{full}}}}}} - \frac{{{{{\mathrm{rank}}}}\left( {{{{\mathbf{C}}}}_{{{{\mathrm{full}}}}}} \right)}}{n}}}} \right)\left( {\frac{{n - {{{\mathrm{rank}}}}\left( {{{{\mathbf{C}}}}_{{{{\mathrm{full}}}}}} \right)}}{{n - {{{\mathrm{rank}}}}\left( {{{{\mathbf{C}}}}_{{{{\mathrm{reduced}}}}}} \right)}}} \right)$$

The metric $\pi _c$, a ratio of estimated expression-mediated causal effects, is obtained by replacing the GWAS effect size vector $\widehat {\theta _ \ast }$ by the effect size vector $\hat \alpha \widehat {\beta _ \ast }$ which quantifies the effect on the trait mediated by the gene expression. Correspondingly, one can estimate the concentration of expression-mediated heritability, *ψ*_*e*_ (see above for definition of *ψ*). The difference vector:

25 $$\widehat {h_ \ast } = \widehat {\theta _ \ast } - \hat \alpha \widehat {\beta _ \ast }$$

is an overall estimate of ‘indirect’ effect, including heterogeneity, confounding, and other non-expression-mediated effect.

### Training the full model and reduced model of gene expression

We generated a “reduced model” (trained using only the subset of variants in the segment of interest) and the “full model” (trained using all variants in the cis-region, 1 Mb on both sides from the gene body). As an application, for the reduced model, we included only the introgressed variants in the Neanderthal-inherited 49.4 Kb segment, and then estimated the expression variance $h_{g,{{{\mathrm{reduced}}}}}^2$ explained by the model:

26 $$\widehat {h_{g,{{{\mathrm{reduced}}}}}^2} = [{{{\mathrm{cor}}}}\left( {g_{{{{\mathrm{test}}}}},\,\widehat {g_{{{{\mathrm{reduced}}}},{{{\mathrm{test}}}}}}} \right)]^2$$

as the square of the correlation between the predicted expression $\widehat {g_{{{{\mathrm{reduced}}}},{{{\mathrm{test}}}}}}$ and observed expression $g_{{{{\mathrm{test}}}}}$ in a test set. This reduced model facilitates comparison with the original full model.

For the actual implementation, we leveraged whole-genome sequence data and gene expression data from the GTEx v8 data release^13^. The sample size ranges from 70 to 706 across 49 tissues from a total of 838 donors. We used the residual of the normalized expression level^13^ after adjusting for covariates: gender, platform, first five principal components (PCs), and PEER factors for each tissue. The reduced model and the full model were trained using JTI^9^ to improve the prediction performance (the square of the Pearson’s correlation *r* between the predicted expression and the observed expression) by borrowing information across tissues. The training of the full model was as previously described^9^. Briefly, JTI estimates the gene expression profile similarity and the regulatory profile similarity (here, generated from the DNase I hypersensitivity [DHS] sites in the promoter region) for each tissue-tissue pair. The two similarity measures were combined using hyperparameters, which were tuned using five-fold cross validation. For the reduced model, the similarity of the regulatory profile was estimated using the DHS peaks in the introgressed segment^41,42^. Genes with a good prediction quality from 5-fold cross-validation (*r* > 0.1 and *P* < 0.05 for the correlation between the observed and the predicted expression) are called *imputable genes* (iGenes). Common genetic variants (minor allele frequency ≥ 0.05) were used for training the full and reduced models. Models trained by PrediXcan and by JTI, and similarly the reduced model and the full model, were systematically compared for prediction quality.

We also compared the prediction performance (*r*^*2*^) of a randomly-chosen segment with that of the actual introgressed segment. For each gene located within 1 Mb of the introgressed segment (in both directions), we built a prediction model for each of 100 randomly-selected segments (of the same length as the introgressed segment) within the *cis*-region (i.e., within 1 Mb of the gene), using the genetic variants in the segment. The median of the prediction performance (*r*^*2*^) across the 100 models was calculated for each gene as the random-segment-based prediction performance.

We investigated the extent to which maintenance of good prediction accuracy with the reduced model (relative to the full model) depended on the segment length. We tested two segment lengths (i.e., 100 and 500 kb extensions on both sides of the actual segment), to compare the performance of the reduced model from the dilated segment and that of the full model from the complete *cis*-region.

### GWAS summary-statistics-based JTI of COVID-19 hospitalization and severity

To identify the genes associated with COVID-19 severity, we applied JTI to the summary statistics from COVID-19 HGI GWAS meta-analyses round 6^6^. For the GWAS meta-analysis of COVID-19 hospitalization, 24,274 hospitalized cases and 2,061,529 population controls were included. The GWAS meta-analysis of severity included 8,779 very severe respiratory confirmed cases and 1,001,875 population controls. Details of each sub-study can be found in Supplementary Table 3.

### Causal gene mapping using Mendelian randomization

Based on the JTI results, we further performed Mendelian randomization to map causal genes around the introgressed segment. Here we applied our MR-JTI^9^ approach, which, through modeling the heterogeneity (from horizontal pleiotropy and unobserved confounding factor) of instrumental variables (IVs), provides a nearly unbiased estimate of the gene causal effect *ɑ* on the trait. To confirm this, we performed simulations (*n* = 500), comparing MR-JTI’s estimate of the causal effect with the conventional inverse-variance weighted (IVW) method’s estimate. We randomly sampled 100 genes with at least one eQTL (estimated from 670 whole blood GTEx v8 samples). The gene expression level (*X*) was simulated using empirical eQTL effect sizes (*β*). The variance of the residual component ($\sigma _X^2$) was also informed by empirical data. The trait (*Y*) was simulated by assuming that the gene expression level was causal for the trait at various levels of effect size *α* (ranging from 0 to 0.5). To investigate the impact of heterogeneity on the causal effect estimate from MR-JTI ($\widehat {\alpha _{{{{\mathrm{JTI}}}}}}$) and IVW ($\widehat {\alpha _{{{{\mathrm{IVW}}}}}}$), we assumed that 20% of the instrumental variables were not valid, with the horizontal-pleiotropy effect (*Z*) twice as large as the mediation effect. For each simulation, the genotype data (*G*) was generated for 50,000 samples based on empirical genotype data (GTEx v8)^13,40^.

27 $$X = \beta G + \varepsilon _X,{\rm{where}}\, \varepsilon _X\sim {{{\mathcal{N}}}}(0,\sigma _X^2)$$

28 $$Y = \alpha X + Z + \varepsilon _Y, {\rm{where}} \, \varepsilon _Y\sim {{{\mathcal{N}}}}\left( {0,1} \right)$$

MR-JTI solves the following optimization problem:

29 $$\hat \alpha ,\hat \delta,\hat \omega = \mathop {{{{{\mathrm{argmin}}}}}}\limits_{u,v_j,w} \mathop {\sum }\limits_{j = 1}^J \left( {\widehat {\theta _j} - u\widehat {\beta _j} - v_j - wl_j} \right)^2 + \lambda \left(\Vert {\left[ {v_j} \right]\Vert_{1} + \left| u \right| + \left| w \right|} \right)$$

to estimate the gene causal effect ($\hat \alpha$), the contribution ($\widehat {\delta _j}$) of the *j*th instrument to the heterogeneity, and the effect ($\hat \omega$) of the LD score *l*_*j*_. Here, $\hat \theta$ is the GWAS effect size vector. MR-JTI is a two-sample Mendelian randomization approach. For implementation, the GTEx v8 eQTL dataset^13^ ($\widehat {\beta _j}$) and the GWAS summary statistics ($\widehat {\theta _j}$) were used as input. The LD score was estimated from GTEx v8 (the same dataset as used for eQTL estimation). For additional support, we also applied MR-Egger and weighted median estimator to estimate the causal effect for each gene using the R package ‘MendelianRandomization’. Following the Mendelian randomization guidelines^43^, we removed palindromic IVs and clumped IVs using PLINK1.9 (--clump-p1 0.05 --clump-r2 0.1) based on the p-value of the association test between an IV and gene expression level. Additional correlation among the IVs was removed by incorporating the IV-IV correlation matrix in the ‘MendelianRandomization’ implementation.

### Genetically determined expression score in modern human populations and an archaic genome

We define the GDE-score of a subject for a gene using the gene’s JTI model^9^. The GDE-score provides a metric to quantify “regulatory divergence” between modern human genomes and an archaic genome, which can be used to investigate phenotypic divergence among hominin lineages^11^ or among individuals according to introgression status. We note that the GDE-score should not be viewed as an extinct hominin’s level of gene expression, which is not directly accessible. The GDE-score does not reflect fixed differences or substitutions, but models only polymorphisms that arose in the common ancestors of modern humans and the archaic genome as well as modern-human specific polymorphisms at which the archaic genome is homozygous for the ancestral alleles^16^. Differences in a gene’s GDE-score quantify differences in genetic regulatory effects for these modeled variants. As an application, we estimated the phenotypic consequence of the introgressed segment for putatively causal genes from the Mendelian randomization analyses.

As a reference panel of modern human populations, individual-level genotype data were downloaded from the 1000 Genomes project (phase 3)^44^. The distributions of estimated genetically determined expression in five populations, including African Ancestry (AFR), American Ancestry (AMR), East Asian Ancestry (EAS), European Ancestry (EUR), and South Asian Ancestry (SAS), were generated. The high-quality archaic genome from a Neanderthal individual found in the Altai Mountains was used to estimate the archaic genome GDE-score^11,45^.

### Identifying the phenomic consequences of a genomic segment

To determine the health consequences of the target genes of the segment, we conducted phenome-wide association studies (PheWAS)^46–48^. We selected genes based on the prediction performance of the reduced model, as these genes show substantial genetic control by the segment in at least one tissue, but we used the full model to evaluate their phenotypic consequences in PheWAS, as the full model should have improved power for the association test.

We performed JTI association analyses on blood cell traits, using the GWAS summary statistics from the UK Biobank samples. The GWAS summary statistics were downloaded from the Neale Lab (www.nealelab.is/uk-biobank). The sample size for the 27 blood cell traits ranges from 344,728 to 350,470. The links for the resource, including the summary statistics and the original distributions of all blood cell traits, can be found in Supplementary Table 10. The covariates age, age^2^, sex, age*sex, sex*age^2^, and the first 20 PCs were considered as covariates in the GWAS.

To identify potential complications of severe COVID-19, we performed a JTI-based phenome scan across four trait categories, specifically neurological, respiratory, circulatory, and endocrine/metabolic disorders, based on the UKB GWAS results. The GWAS summary statistics had been generated by the Lee lab^49^, using SAIGE (Scalable and Accurate Implementation of GEneralized mixed model), which provides accurate *P* values even when case-control ratios are extremely unbalanced^50^. In total, 253 binary traits (belonging to the four categories) with at least 50 cases were included. The first four genotype-based principal components, gender, and birth year were included as non-genetic covariates. The Phecode hierarchical system (https://phewascatalog.org/)^51,52^ comes with case groups (typically diseases and complications), each with a corresponding control group. The sample size for each trait can be found in Supplementary Table 12.

### Reporting summary

Further information on research design is available in the Nature Research Reporting Summary linked to this article.

## Supplementary information

Supplementary_tables

Supplementary_Materials

Reporting Summary

## Data Availability

The COVID-19 severity GWAS summary statistics are publically accessible. The JTI prediction models are available at https://zenodo.org/record/3842289.

## Code availability

The code for SEGMENT-SCAN is available at Github (https://github.com/gamazonlab/DeepRegionalAnalysis).
