# Supplementary material for: Integrative transcriptomic, evolutionary, and causal inference framework for region-level analysis: Application to COVID-19
Source: NPJ Genom Med. 2022 Mar 22;7:24. doi: 10.1038/s41525-022-00296-y (PMC8940898; doi:10.1038/s41525-022-00296-y)
Supplement: Supplementary file 2 — Supplementary_Materials [file 41525_2022_296_MOESM2_ESM.pdf]

Supplementary Materials for

Integrative transcriptomic, evolutionary, and causal inference framework for region-level analysis: Application to COVID-19

Dan Zhou and Eric R. Gamazon

Send correspondence to: Eric R. Gamazon ericgamazon@gmail.com

This PDF file includes:

Supplementary Figures 1 to 6.
References (1)

Other Supplementary Materials for this manuscript include the following:

Supplementary Table 1 to 13 (included in excel spreadsheets)

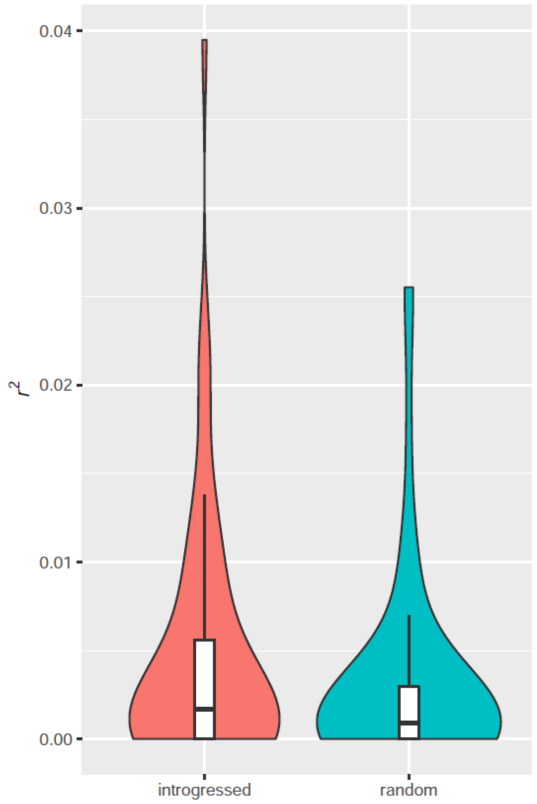

Supplementary Figure 1. The introgressed segment carries more regulatory information than a randomly-chosen segment. For each gene located within 1 Mb of the introgressed segment (on both sides), we built a prediction model for each of 100 randomly-selected segments, using the SNPs in the segment as features. The median of the prediction performance (r^2) across the 100 models was calculated for each gene and was defined as the prediction performance of a randomly-chosen segment. The distributions of the prediction performance across all the genes in the region from the actual introgressed segment and, for comparison, from the randomly-chosen segments are shown in the violin plot and the boxplot. The median of the proportion is visualized as a black segment in the middle of the box. The lower and upper hinges correspond to the first and third quartiles (the 25th and 75th percentiles). The upper / lower whisker extends from the hinge to the largest / smallest value no further than / at most 1.5 * IQR from the hinge (where IQR is the inter-quartile range or the distance between the first and third quartiles). In this analysis, we pooled the prediction models estimated from lung and whole blood and compared the performance.

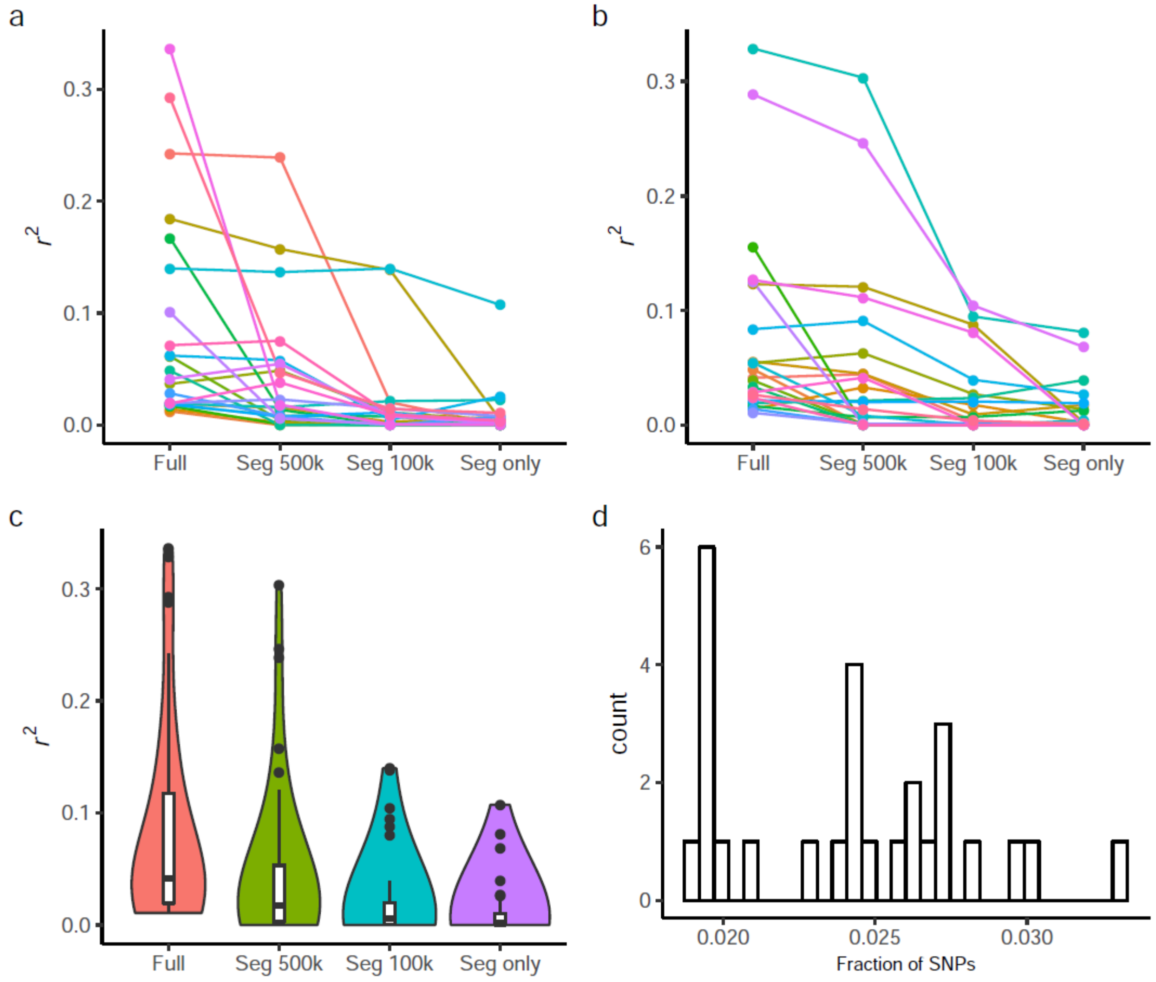

Supplementary Figure 2. The introgressed segment accounts for a substantial proportion of regulatory information, which varies by gene and tissue. Prediction performance (r^2) comparison for four different input segment lengths for model building. "Full" denotes the model that includes all the SNPs within 1 Mb of the gene body (on each side). "Seg 500k" and "Seg 100k" denote models that include the SNPs within 500 kb and 100 kb of the introgressed segment (on each side), respectively. Note the SNPs not overlapping with the full model region were not included. The "Seg only" denotes a model that includes only the SNPs in the segment. i.e., the so-called "reduced model". To show the decrease in the prediction performance, we marked each gene with its own color in panel **a** (lung tissue) and **b** (whole blood). A comparison of the distribution of the prediction performance (across genes and tissues) is shown in panel **c**. The distribution of the proportion of the SNPs in the segment (i.e., the number of SNPs in the segment divided by the number of SNPs within the full 1 Mb region) is shown in panel **d**.

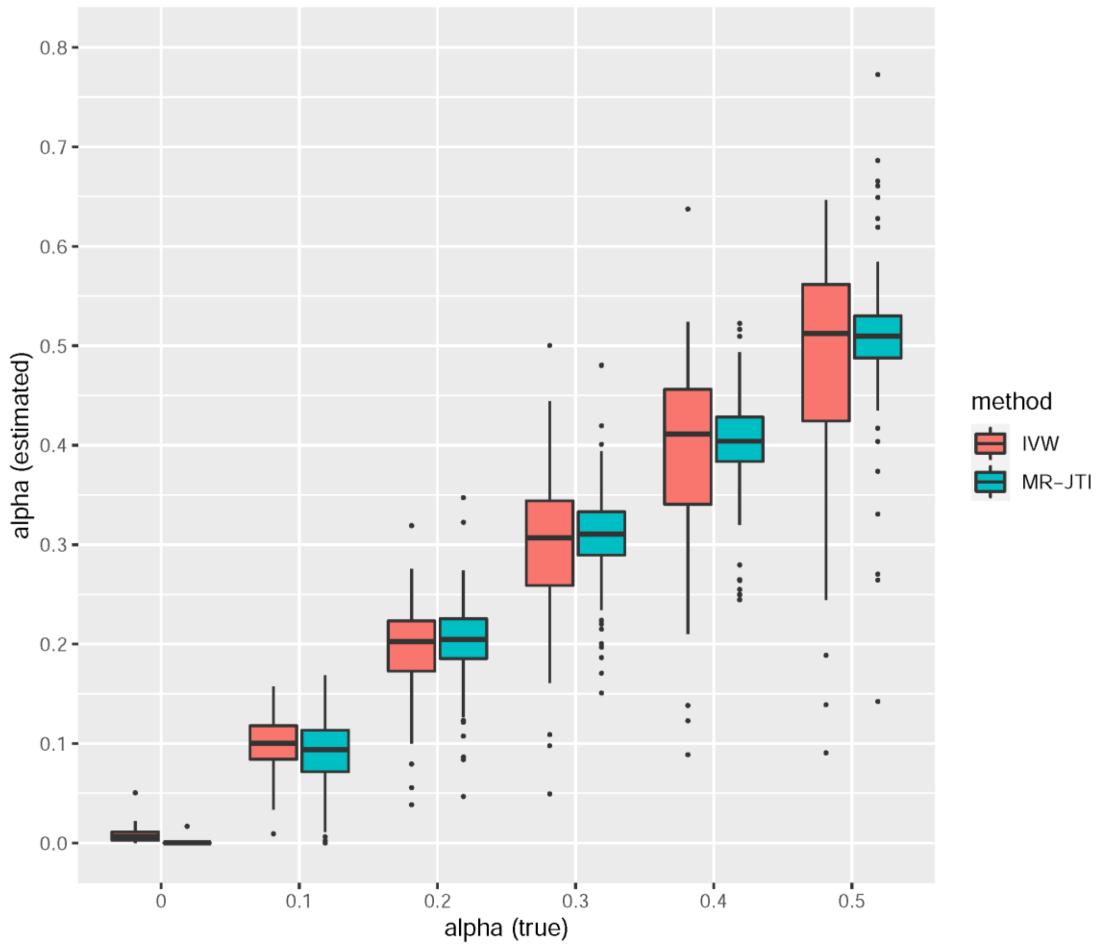

Supplementary Figure 3. MR-JTI provides an approximately unbiased estimate of the effect of gene expression on outcome. Through extensive simulations, we compared the estimate of the effect size of gene expression on trait, when the true effect size (alpha) was varied from 0 to 0.5, between the MR-JTI method and the IVW (inverse-variance weighted) approach. The boxplot shows the distribution of the estimate of the effect size of gene expression on the trait. The median of the proportion is visualized as a black segment in the middle of the box. The lower and upper hinges correspond to the first and third quartiles (the 25th and 75th percentiles). The upper / lower whisker extends from the hinge to the largest / smallest value no further than / at most 1.5 * IQR from the hinge (where IQR is the inter-quartile range or the distance between the first and third quartiles). MR-JTI provides an approximately unbiased estimate of the effect size. Notably, the IVW estimator, which does not account for the pleiotropy effect, has a generally higher variance, which becomes more pronounced as a larger true effect size is assumed.

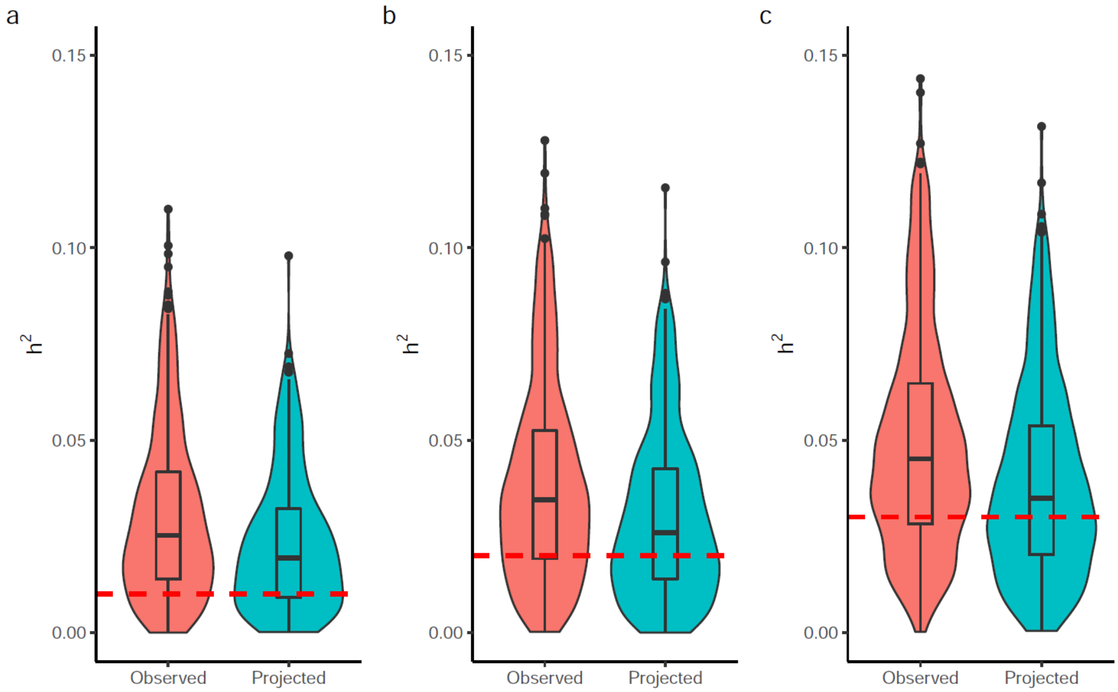

Supplementary Figure 4. The estimated local heritability using the projected LD matrix is less biased than the corresponding estimate using the observed LD matrix. Simulations were performed to compare the estimate of heritability calculated using the observed LD matrix and the projected LD matrix. The distribution of the estimated local heritability, i.e., attributable to a segment, (across 500 simulations) is shown in a violin plot and a boxplot. The median local heritability is visualized as a black segment in the middle of the box. The lower and upper hinges correspond to the first and third quartiles (the 25th and 75th percentiles). The upper / lower whisker extends from the hinge to the largest / smallest value no further than / at most 1.5 * IQR from the hinge (where IQR is the inter-quartile range or the distance between the first and third quartiles). The estimated heritability calculated using the projected LD matrix is closer to the ground truth (**a** 0.01, **b** 0.02, and **c** 0.03, marked as a red dashed line) than the estimated heritability using the observed LD matrix.

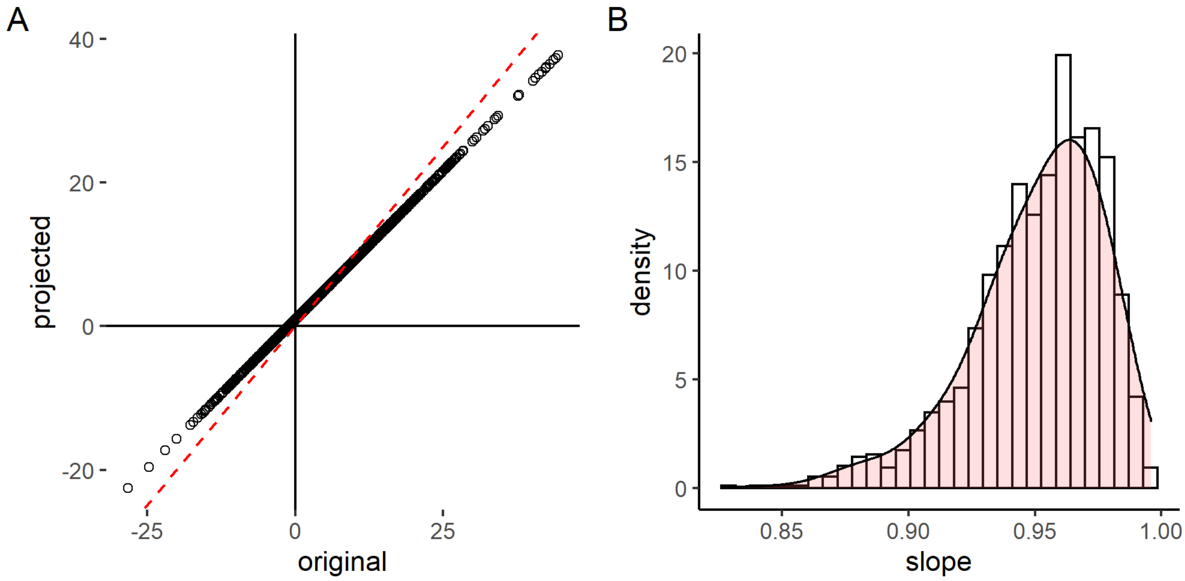

Supplementary Figure 5. Row sum comparison between the original and projected LD matrix indicates inflation in the summary-statistics-based heritability estimate based on the original LD matrix as the reference. Panel (**A**) shows an example obtained by regressing the row sum of the projected SNP-SNP covariance matrix (LD matrix) on the row sum of the original (observed) LD matrix. Here, the genotype dosage was scaled to the standard normal distribution $\mathcal{N}(0,1)$. The projection was performed using Ledoit-Wolf shrinkage (by implementing the function 'covEstimation' from R package 'RiskPortfolios'). The slope of the regression line is less than 1 (i.e., the slope of the red dash line $y = x$). The distribution of the slope among all autosomal LD blocks is shown in panel (**B**). The putative LD blocks were generated by Berisa et al[1]. Note that for most LD blocks on the autosomes, the slope is less than 1, which would produce inflated heritability estimates based on the original LD matrix as the reference.

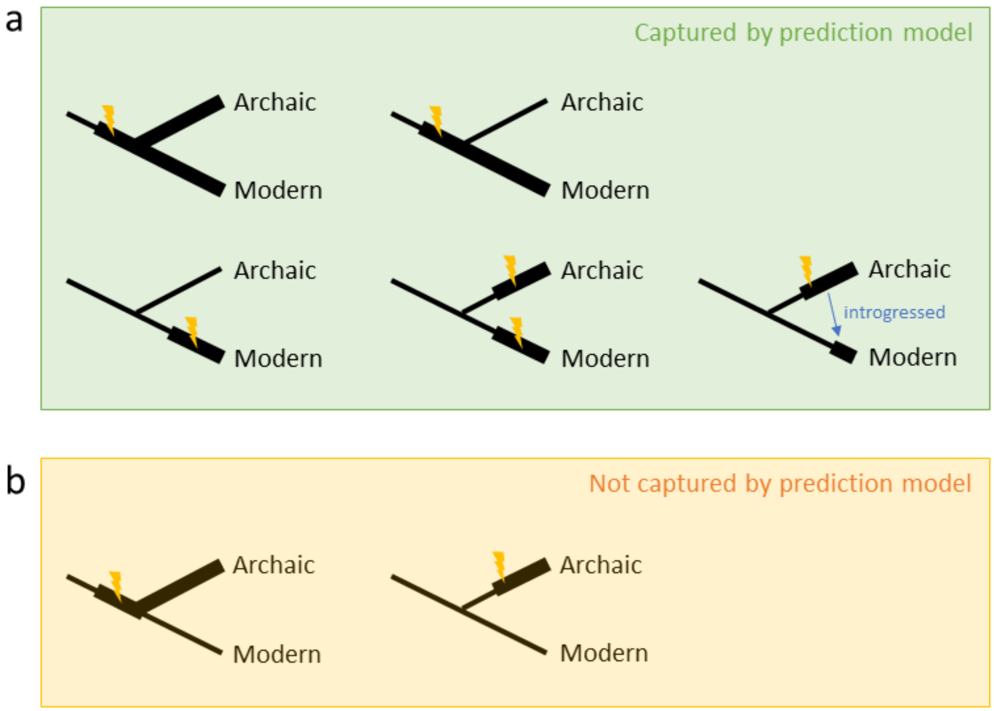

Supplementary Figure 6. Categories of genetic variants included in the gene expression prediction models. JTI (or the earlier PrediXcan) predicts gene expression in modern human populations using variants that can be categorized according to their evolutionary histories (panel a). The different categories of variants that can be modeled are: (1) variants ancestral to the 'archaic' profile and modern humans and that remain polymorphic in modern humans and in the archaic profile, (2) variants ancestral to the 'archaic profile' and moderns humans and that became fixed in the archaic profile, (3) variants specific to modern humans, (4) variants that occurred on each lineage independently, and (5) variants that appeared in the archaic lineage and were introgressed into modern humans. The framework cannot directly model the effects of archaic-lineage-specific variants, which have become fixed in the modern human lineage or have not been introgressed into modern humans.

Reference

1 Berisa, T. & Pickrell, J. K. Approximately independent linkage disequilibrium blocks in human populations. *Bioinformatics* **32**, 283 (2016).
